# Identification of CSP Types and Genotypic Variability of Clinical and Environmental Isolates of *Aspergillus fumigatus* from Different Geographic Origins

**DOI:** 10.3390/microorganisms8050688

**Published:** 2020-05-08

**Authors:** Esperanza Duarte-Escalante, María Guadalupe Frías-De-León, Erick Martínez-Herrera, Gustavo Acosta-Altamirano, Emmanuel Rosas de Paz, Jesús Reséndiz-Sánchez, Nicolás Refojo, María del Rocío Reyes-Montes

**Affiliations:** 1 Departamento de Microbiología y Parasitología, Facultad de Medicina, Universidad Nacional Autónoma de México (UNAM), Ciudad Universitaria No. 3000, Mexico Cd. Mx. 04510, Mexico; dupe@unam.mx (E.D.-E.); emmrodepaz@gmail.com (E.R.d.P.); 2 Hospital Regional de Alta Especialidad de Ixtapaluca, Carretera Federal México-Puebla Km. 34.5, Pueblo de Zoquiapan, Ixtapaluca 56530, Mexico; magpefrias@gmail.com (M.G.F.-D.-L.); erickmartinez_69@hotmail.com (E.M.-H.); mq9903@live.com.mx (G.A.-A.); 3 Laboratorio de Micología, Hospital Infantil de México “Federico Gómez”, Doctor Márquez 162, Mexico Cd. Mx. 06720, Mexico; elmico2000@yahoo.com.mx; 4 Departamento de Micología, Instituto Nacional de Enfermedades Infecciosas ‘Dr. Carlos G. Malbrán’, Buenos Aires C1282AFF, Argentina; nrefojo@hotmail.com

**Keywords:** cell surface protein, microsatellite marker, typing

## Abstract

The CSP (cell surface protein) microsatellite marker is useful for typing *Aspergillus fumigatus* isolates and determining relationships at the subpopulation level because it has shown high discriminatory power. In the present study, 90 *A. fumigatus* isolates from Mexico (MX), Argentina (AR), France (FR), and Peru (PE) were identified through a phylogenetic analysis using the *benA* gene fragment and were typed with the CSP microsatellite, and the types were identified using the nomenclature recommended in the literature. Genetic variability was analyzed through haplotype diversity, nucleotide diversity, polymorphic sites, and nucleotide differences between pairs of sequences. The population structure was evaluated using the Tajima’s D statistic. No new CSP types were recorded in the MX, FR, and PE isolates, while in the AR isolates, two new CSP types were identified (t25 and t26). The most common CSP types in the studied populations were t01, t02, t03, and t04A; these results are consistent with findings in other countries. In addition, the genetic diversity parameters we obtained revealed that the greatest genetic diversity was found in the MX population, followed by AR and FR. No population structure was identified among the isolates studied.

## 1. Introduction

Aspergillosis is caused by fungi of the genus *Aspergillus*, which contains etiological agents that are capable of causing different clinical forms of the disease in humans, of which invasive aspergillosis is the most severe form [1]. This clinical form has increased dramatically over the past two decades and is often fatal, despite diagnostic and therapeutic efforts [2], and an increase in resistance to the antifungals commonly used as therapy for this disease has been observed [3]. In addition, in recent years, new species have been identified within the genus *Aspergillus* that can cause infections in humans [4].

Currently, the classification of the genus *Aspergillus* consists of four subgenera: *Aspergillus* (with two sections), *Circumdati* (with six sections), *Fumigati* (with three sections), and *Nidulantes* (with 10 sections) [5,6], with a total of 339 accepted species [7]; however, cryptic species continue to be detected that are difficult to differentiate from related species. For this reason, PCR-based molecular methods are used, using sequencing of gene fragments such as 18S rRNA, mitochondrial DNA, rodlet A (rodA), β-tubulin (*benA*, *tub-2*), actin (*Act*), calmodulin (*caM*), and internal transcribed spacer regions (ITS) [8,9] for identification at the species level. The ISHAM (International Society for Human and Animal Mycology) reports that the sequence analysis of the rDNA internal transcribed spacer region ITS1-5.8S-ITS2 is particularly used for the clinical identification of fungi; however, these regions are not sufficiently polymorphic; therefore, they are only useful for identifying *Aspergillus* at the section level. However, for identification at the species level, other markers such as *benA*, *tub-2*, and *caM*, which are crucial genes in the *Aspergillus* taxonomy, can be used in combination with the ITS region [10].

Given the increasing frequency of *Aspergillus* species associated with disease in humans and their resistance to antifungals, it is important to identify them at the species level to provide appropriate treatment to patients with aspergillosis [11]. In addition, it is important to know the molecular distribution and epidemiology of *Aspergillus* species obtained from clinical and environmental sources in different geographic regions of the world. To obtain information on different aspects of the disease and the etiological agents that cause it, molecular epidemiology studies were carried out using different molecular methods [12,13,14,15,16]. Among these methods is a very accurate typing method that involves a 12-mer microsatellite found in the region coding for a putative cell surface protein (CSP) (AFUA_3G08990); this method was used to type *A. fumigatus* isolates obtained from nosocomial outbreaks of invasive aspergillosis, and is able to demonstrate clonality and different genotypes among the isolates [17]. Additionally, the results using this method were consistent with those obtained using *Afut1* RFLP (*Afut1* restriction fragment length polymorphism) [18], which has high discriminatory power [17]. As such, typing with the CSP marker is an alternative to distinguish *A. fumigatus* isolates, because the diversity of sequence types found within the repeated region of the CSP gene is much higher than that previously reported with other markers. Taking into account the characteristics of the CSP marker, Klaassen et al. [19] subtyped a collection of clinical isolates of *A. fumigatus* recovered from several hospitals and identified CSP lineages of closely related types within the isolates studied. They also proposed nomenclature for individual repeats, as well as for CSP types, to normalize the presence of CSP types distributed throughout the world. Several researchers [20,21,22] have used the same strategy proposed by Klaassen et al. [19] to type clinical and environmental isolates from Australia, China, and Iran, to find phylogeographic differences with isolates from Europe and North America, and their findings showed that some CSP types are shared; however, they reported new variants in isolates from Australia and China.

Currently, in Latin America, there are no studies that provide evidence of the usefulness of this method in the subspecific typing of clinical and environmental isolates of *A. fumigatus,* nor was any method routinely implemented in the study of nosocomial outbreaks. Therefore, in this study, *A. fumigatus* species of clinical and environmental isolates from different institutions in Mexico (MX), Argentina (AR), France (FR), and Peru (PE) were identified and typed by analyzing the sequences of the CSP microsatellite. Additionally, the sequences obtained were analyzed according to the method of Klaassen et al. [19].

## 2. Materials and Methods

### 2.1. Fungal Isolates

A total of 90 fungal isolates, clinical and environmental, from the *Fumigati* section of the genus *Aspergillus* were included in the study and identified (Table 1). The reference strains *A. fumigatus* (ATCC MYA-3626) and *A. lentulus* (ATCC MYA-3566) were also included. Isolates were obtained from the Collection of the Laboratory of Molecular Mycology, Department of Microbiology and Parasitology, School of Medicine, National Autonomous University of Mexico (UNAM) and the Laboratory of Mycelial Fungi of the Department of Mycology of the National Institute of Infectious Diseases–National Administration of Health Laboratories and Institutes (INEI–ANLIS) (“Dr. Carlos G. Malbrán”, Argentina, Argentina). Isolates were cultured at 28 °C in potato dextrose agar (PDA) (Bioxon, CDMX, Mexico).

### 2.2. Obtaining Monosporic Cultures

From each isolate cultured in PDA (Bioxon, CDMX, Mexico) for 2–4 days at 28 °C, a conidia suspension was prepared with 1 mL of phosphate buffer (pH 7.4) and 0.05% Tween 20 (PBST). This suspension was diluted to 1:1000 with PBST, and 50 μL was taken and inoculated in Petri dishes with PDA and incubated at 28 °C. An isolated colony was selected from each plate and cultured in a tube with the same medium at 28 °C. Conidia of monosporic cultures were preserved in sterile water at 4 °C.

### 2.3. Macromorphology and Micromorphology

The colonial morphology of each isolate was analyzed from PDA cultures at 28 °C and incubated for 4–7 days. The micromorphological characteristics of the isolates of *Aspergillus* spp. were analyzed using Riddell’s microculture method [23]. The microculture was incubated at 37 °C for 4 days, or until the fungus was observed to grow. Subsequently, the cover slip was carefully separated from the agar, placed on a slide with a drop of cotton blue stain, and observed under the microscope. We analyzed the morphological characteristics using a microscope and a drop of cotton blue stain. The microscopic characteristics of the isolates were recorded using a digital camera.

### 2.4. DNA Extraction

From each monosporic culture of *Aspergillus* spp. seeded in PDA (Bioxon, CDMX, Mexico), a conidial suspension was obtained in 1 mL phosphate buffer pH 7.5 supplemented with Tween 80, inoculated in 50 mL of YEPG culture medium (1% yeast extract, 2% peptone, 2% dextrose), and incubated at 37 °C under agitation for 2 days or until mycelium growth was observed. The mycelial biomass of each isolate was harvested by filtration through Whatman No. 1 filter paper. The mycelium DNA was extracted using a DNeasy^®^ Plant Mini Kit (Qiagen, Austin, TX, USA), as described by Refojo et al. [16]. DNA concentration and purity (absorption ratio 260/280 = 1.8–2.0) were determined in a DS-11 spectrophotometer (DeNovix, Wilmington, DE, USA).

### 2.5. Amplification of a Fragment of the β-Tubulin Gene (benA)

The amplification of the *benA* gene fragment was carried out using the oligonucleotides Bt2a (5´-ggtaaccaaatcggtgctgctttc-3´) and Bt2b (5´-accctcagtgtagtgacccttggc-3´), as described by Glass and Donaldson [8]. The reactions were carried out in an ESCO Swift^®^Maxi^TM^ ThermalCycler Block thermocycler (Esco Healthcare PTE. Ltd., Singapore, SG). The amplicons were separated on 2% agarose gels stained with GelRed (10,000×, Biotium Inc., Fremont, CA, USA), in 0.5× TBE buffer (45 mM Tris-Base, 45 mM boric acid, 1 mM EDTA), at 100 V. A 100 bp DNA ladder (molecular size marker) (Invitrogen, Carlsbad, CA, USA) was used. The gels were visualized in a Synoptics Photodocumenter (Syngene, Frederick, MA, USA), using the program GeneSnap (Syngene, Frederick, MA, USA) ver. 6.03.00. The amplicons obtained were sequenced in both directions by the Sanger method in Macrogen USA (Rockville, MD, USA), and the sequences were deposited in the GenBank database (http://www.ncbi.nlm.nih.gov), under the accession numbers (MN637695–MN637781).

### 2.6. Sequence Analysis benA Gene Fragment

The program Bioedit ver. 7.1.9. (www.mbio.ncsu.edu/BioEdit/bioedit.html) was used, which allowed us to manually check the sequences obtained during the sequencing process (forward and reverse) for each isolate and also create a consensus sequence. Each of the sequences was analyzed with the program BLAST (Basic Local Alignment Search Tool) [24] (www.blast.ncbi.nlm.nih.gov/blast.cgi) to verify its identity. Subsequently, the sequences were aligned with the MAFFT program (http://mafft.cbrc.jp/alignment/server/) [25], and the best evolutionary model applied to this alignment was chosen with the program JModelTest 2 (www.github.com./ddarriba/jmodeltest2) [26].

### 2.7. Phylogenetic Analysis benA Gene Fragment

For the phylogenetic analysis, 25 reference sequences corresponding to the sequences of the *benA* gene of isolates of the genus *Aspergillus* were used; the sequences were obtained from GenBank (Table 2).

The maximum likelihood method was used to identify *A. fumigatus*. A phylogeny test was used that included the bootstrap method with 1000 replicates and the GTR G + I evolutionary model; the NNI (nearest neighbor interchange) heuristic method was applied, and analysis was carried out with the program MEGA 6 [27].

### 2.8. Amplification of the CSP Microsatellite

Isolates identified as *A. fumigatus* were selected for genotyping based on the sequence of the CSP microsatellite. The amplification of this sequence was performed using the oligonucleotides CSP-F (5´-TTGGGTGGCATTGTGCCAA-3´) and CSP-R (5´-GGAGGAACAGTGCTGTTGGTGA-3´), which amplified fragments ~550 to 700 bp [28]. The reaction mixture (25 μL) consisted of 5 ng of DNA, 200 μM dNTPs, 1.2 mM MgCl_2_, 100 pmol of each CSP-F and CSP-R oligonucleotide (Sigma–Aldrich, St. Louis, MO, USA), and 1 U of *Taq* polymerase (Invitrogen Life Technologies, Carlsbad, CA, USA), in 1× buffer. The amplification was performed in an ESCO^®^ Swift Maxi thermocycler (Micro Pte Ltd. ver. 1.0) according to the program reported by Balajee et al. [17]: one 5 min cycle at 94 °C followed by 35 cycles of 15 s at 94 °C, 30 s at 55 °C, and 30 s at 68 °C, and a final extension of 2 min at 68 °C. The amplification products were analyzed by electrophoresis in 1.5% agarose gels (Pronadisa, MD, ES) in 0.5× TBE buffer stained with GelRed (10,000×, Biotium Inc.). The standard molecular size used was a 100 bp DNA ladder (Invitrogen Life Technologies). Images of the gels were captured in a Synoptics Photodocumenter (Syngene, Frederick, MA, USA). The amplified fragments were purified using a QIAquick Gel Extraction Kit (Qiagen), following the instructions provided by the manufacturer. Subsequently, the fragments were sequenced by the Sanger method in Macrogen (Rockville, MD, USA).

### 2.9. CSP Microsatellite Sequence Analysis

The CSP sequences were also verified through the program BioEdit v7.0.9 to create a consensus sequence for each isolate. Repetition types and types of CSP sequences were assigned to the studied isolates. Repetition types were identified from the region of the CSP gene sequence and were assigned with a random number; 10 repeat type sequences are currently known (Klaassen et al. [19] and Kidd et al. [20]) (Table 3). The CSP type is built upon the identification of the repeat types in the region of the CSP gene sequence and, based on the differences in the number of repeats, they were assigned to the already identified CSP types or to newly identified types.

### 2.10. Genetic Diversity

The genetic diversity was estimated through two parameters: haplotype diversity (Hd = probability that two randomly selected haplotypes of a sample are different) and nucleotide diversity (Pi = average number of nucleotide differences per site between two sequences chosen at random), the number of segregated sites or polymorphic sites (S), and the average number of nucleotide differences between pairs of sequences (K)) using the program DnaSP version 5 [29].

The neutrality model was used, which has a null hypothesis that the molecular variation is the result of a balance between neutral or random mutations and the extinction of mutations by genetic drift, maintaining the genetic frequencies in equilibrium [30,31]. To search for deviations from this model, we used the D [32], D* [33], and FS [34] statistics. The level of statistical significance of the three estimates was evaluated with 10,000 repetitions (with coalescence simulation) in DnaSP version 5 [29].

## 3. Results

### 3.1. Phenotypic and Molecular Identification of A. fumigatus

The 90 isolates were identified as *A. fumigatus* according to their phenotypic characteristics, and they exhibited macroscopic and microscopic morphological characteristics reported for the *Fumigati* section. The texture of the colonies was velvety or powdery, the front color was teal with a white border, and the back was cream-colored. Microscopic morphology of the isolates showed vesicles that were subclavate in shape, and flask-shaped conidiogenous cells that were uniseriate and compact, usually forming on the upper two-thirds of the vesicle.

To determine *A. fumigatus* species, a phylogenetic analysis was performed with the sequences of the *benA* gene fragment from all isolates studied. All isolates were grouped with the reference strains corresponding to *A. fumigatus* with a 96–99% bootstrap, and were identified as *A. fumigatus* (Supplementary Figure S1).

Newly generated sequences from this study were deposited in GenBank/EMBL (European Molecular Biology Laboratory) databases under the provisional accession numbers (MN637695–MN637781).

Using the nomenclature proposed by Klaassen et al. [19] and modified by Kidd et al. [20] and Gao et al. [21], the 90 isolates of *A. fumigatus* were typed. From the analysis of the sequences of DNA within the tandem repeats, as well as the flanking regions of –45 bp and +9 bp, we identified a total of 10 CSP types (t01, t02, t03, t04A, t06B, t10, t13, t14, t25, and t26) that are shown together with all CSP variants recognized in previous studies (Table 4).

In isolates from MX, FR, and PE, no new CSP types were recorded; however, in isolates from AR, two new CSP types (t25 and t26) were identified that were not previously described in isolates from the Netherlands, North America, Australia, China, and Iran [17,19,20,21,22]. The t25 CSP type showed 11 tandem repeats, while t26 and t20, with four tandem repeats, have the shortest repeats observed to date.

In Table 5, the prevalence of the CSP types obtained in the different geographic areas studied is shown. The most common CSP types were t01, t02, t03, t04A, t06A, t06B, t10, t13, and t14, and these were also the most prevalent in previous studies; however, the t06A and t06B types were not observed in AR. In FR, only types t03 and t04A were observed, and in PE, only types t01 and t04A were identified. In addition, the types t10, t13, and t14 were less prevalent and were also present in the Netherlands, Australia, and China; however, type t10 and t13 did not occur in AR, FR, and PE, and t14 was not present in FR or PE. In addition, CSP types t15, t16, t17, t18A, t18B, t19, t20, t21, t22, t23, and t24 did not occur in MX, AR, FR, or PE.

### 3.2. Genetic Diversity

The parameters of genetic diversity (Hd, Pi, and K) calculated for *A. fumigatus* isolates revealed that the greatest genetic diversity was found in MX, followed by AR, and lastly FR. The isolates from Peru were not included because the DnaSP program has a limitation and requires more than three sequences to perform an analysis of groups. Data from the Mexican and Argentinian populations were statistically significant (*p* < 0.001 and *p* < 0.05, respectively); however, the FR isolates showed *p* values > 0.10, which are not statistically significant. On the other hand, the D statistics of Tajima showed negative values for the populations of MX, AR, and FR (Table 6).

## 4. Discussion

The phenotypic identification of isolates of *A. fumigatus* from MX, AR, FR, and PE, included in this study, through their macro- and micromorphology, revealed that all isolates belonged to the *Fumigati* section. Additionally, the phylogenetic analysis with sequences of the *benA* gene region confirmed that all isolates belonged to the species *A. fumigatus*. This region of the β-tubulin gene was shown to be useful for studies of the phylogenetic relationships of *Aspergillus* and related species [35] because it is a conserved, slow-evolving gene with a high degree of interspecific variability in the intronic regions, which is sufficient to distinguish three of the main species within the *Fumigati* section: *A. fumigatus*, *A. lentulus*, and *Neosartorya udagawae* [15]. Furthermore, there are no reference sequences corresponding to all species of the *Fumigati* section deposited in GenBank pertaining to the *caM* markers and ITS regions, while the sequences of the *benA* gene encompass a wide number of species, so this was one of the criteria that was used to compare our sequences with the ones deposited in GenBank. Moreover, the *benA* marker was used in other works to identify the *Aspergillus* species, which allows for a comparative analysis.

The typing of the 90 isolates of *A. fumigatus* from MX, AR, FR, and PE using the CSP microsatellite, which was shown to be 100% efficient in previous studies [21], represents the first report of CSP types in these countries. All isolates of *A. fumigatus* could be typed using the CSP microsatellite, and the results showed that the CSP types t01, t02, t03, and t04A predominate in the studied populations of MX and AR, as well as in the United States, Netherlands, Australia, China, and Iran, while the rest of the CSP types are found in low percentages, and some types are country-specific [17,19,20,21]. The presence of CSP types t01, t02, t03, and t04A in the different countries studied suggests that these types are more resistant to environmental pressures, which is why they are the most prevalent types of CSP—perhaps because of an evolved resistance to antifungals or other factors that allow them to survive adverse conditions [36]. On the other hand, the predominance of these types in different Latin American countries suggests the possible introduction of these types through the United States, given that this is the North American country closest to Mexico. This introduction could be the result of frequent migratory movements of humans and by the SAL (Saharan Air Layer), which is an air mass that forms over the Sahara Desert in the spring, summer, and part of autumn, and usually moves towards the North Atlantic Ocean every 3–5 days. This layer can extend vertically between 1500 and 6000 m (5000 to 20,000 feet) in altitude in the troposphere, and is related to large amounts of very dry and dust-laden air (~50% less moisture than typical wet tropical records) that moves with strong winds (25–55 mph or 10–25 m/s). The SAL can cover an area equivalent to the size of North America. Similarly, these masses were traced westward to the Caribbean Sea, Central America, and the Gulf of Mexico. Thus, the Saharan dust could carry various microorganisms, including fungi such as *Aspergillus* (Cooperative Institute for Meteorological Satellite Studies–CIMSS); National Oceanic and Atmospheric Administration–NOAA) (https://cimss.ssec.wisc.edu/).

Likewise, the type t03 was identified in the isolates from MX, AR, and FR, in contrast to the isolates from PE in which this type was not identified. On the other hand, t04A was identified in isolates from MX, AR, FR, and PE, and type t01 was identified in isolates from MX, AR, and PE, but not in isolates from FR. Two new CSP types, t25 and t26, were identified in the isolates from AR. The analysis of CSP types in clinical and environmental isolates from MX and AR showed that there are more CSP types in the clinical isolates than in the environmental isolates. In clinical isolates from MX, eight types (t01, t02, t03, t04A, t06A, t06B, t13, and t14) were identified, while in environmental isolates, only five types (t01, t02, t03, t04A, and t10) were identified; in clinical isolates from Argentina, seven types (t01, t02, t03, t04A, t14, t25, and t26) were identified, and in environmental isolates, only four types (t01, t02, t03, and t04A) were identified. These differences could be accounted for because it was shown that *A. fumigatus* undergoes changes during its adaptation process to the human host, which involves changes at the physiological and genetic levels that allow it to persist in this ecological niche [37]; these changes were identified through SNPs (single nucleotide polymorphisms) in different isolates of the same patient and suggest that these developed during the course of the infection in the host through a microevolutionary process, confirming the plasticity of this fungus. Thus, the findings of this study, related to the fact that more types of microsatellites were found in clinical isolates than in environmental isolates, could be explained by this ability of the fungus to adapt to the host [37].

On the other hand, among the isolates of *A. fumigatus* included in the present study, the isolates from MX showed greater genetic variability because nine CSP types were identified. The presence of different CSP types in a given geographic area may be the result of gene flow because when different populations of the fungus are introduced in a geographic area, mating between members of different populations may lead to hybrids, and these can mate with individuals in any of the parental populations, promoting the introgression of genes from one population to another [38].

Regarding the population structure of this fungus, Tajima’s D statistic showed negative values for the populations of MX, AR, and FR, indicating that these populations experienced a relatively recent demographic population expansion or purifying selection effect [32,39]. This genetic diversity of *A. fumigatus* within the geographic regions studied is consistent with the findings of Pringle et al. [40] for *A. fumigatus,* and the arguments made by Raper et al. [41] who claimed that species of fungi with a wide geographical distribution have little potential to establish genetic structure; therefore, the lack of population genetic differentiation observed globally in this species could be due to a continuous flow of genes across continents because the conidia of this fungus float in the air column, and are protected against UV light due to melanin. They also have tolerance to a wide range of temperatures, which allows them to survive in the air column and the soil. It is important to mention that the presence of genetic variability in this fungus is of great relevance, because it can result in new mechanisms of pathogenesis or resistance to antifungals.

The typing of *A. fumigatus* isolates through the CSP microsatellite in different geographic areas will allow us to understand the prevalence of different types found because, to date, some types remain country-specific. This is important because it reinforces the importance of producing species-specific markers in different geographic regions for diagnosis. In addition, the characterization of genetic diversity will allow us to understand the spread of this fungus and, as a result of spread, understand the changes that the fungus undergoes in different environments that can generate genetic variability.

## 5. Conclusions

To date, 29 types of CSP have been described in populations of *A. fumigatus* from North America, the Netherlands, Australia, China, Iran, Mexico, France, and Peru. These findings corroborate the importance of maintaining continuous surveillance to monitor the genetic diversity of this fungus, as it can have an effect on the emergence of strains resistant to antifungals, in addition to their ability to spread through long-distance dispersion events—another important factor that gives it an opportunity to recombine with populations in diverse geographic regions.

## Figures and Tables

**Table 1:** CSP (cell surface protein) type, geographical origin, and source of *Aspergillus fumigatus* isolates.

Isolate	CSP Type	Geographical Origin	Source
MM-133	t01	MX	Environmental
MM-134	t01	MX	Environmental
MM-131	t01	MX	Environmental
MM-120	t02	MX	Environmental
MM-117	t02	MX	Environmental
MM-08	t02	MX	Environmental
MM-135	t03	MX	Environmental
MM-114	t03	MX	Environmental
MM-121	t03	MX	Environmental
MM-126	t04A	MX	Environmental
MM-116	t04A	MX	Environmental
MM-115	t04A	MX	Environmental
MM-141	t04A	MX	Environmental
MM-118	t04A	MX	Environmental
MM-119	t04A	MX	Environmental
MM-122	t04A	MX	Environmental
MM-139	t04A	MX	Environmental
MM-127	t04A	MX	Environmental
MM-132	t04A	MX	Environmental
MM-140	t04A	MX	Environmental
MM-137	t04A	MX	Environmental
MM-138	t04A	MX	Environmental
MM-124	t04A	MX	Environmental
MM-128	t10	MX	Environmental
MM-129	t10	MX	Environmental
MM-125	t10	MX	Environmental
MM-111	t04A	MX	Clinical
MM-109	t03	MX	Clinical
MM-117	t02	MX	Clinical
MM-33	t02	MX	Clinical
MM-108	t02	MX	Clinical
MM-95	t02	MX	Clinical
MM-46	t01	MX	Clinical
MM-106	t01	MX	Clinical
MM-110	t01	MX	Clinical
MM-136	t01	MX	Clinical
MM-09	t04A	MX	Clinical
MM-10	t04A	MX	Clinical
MM-36	t04A	MX	Clinical
MM-38	t04A	MX	Clinical
MM-45	t04A	MX	Clinical
MM-35	t04A	MX	Clinical
MM-92	t06B	MX	Clinical
MM-99	t06B	MX	Clinical
MM-103	t06B	MX	Clinical
MM-101	t06B	MX	Clinical
MM-100	t06B	MX	Clinical
MM-98	t06B	MX	Clinical
MM-93	t06B	MX	Clinical
MM-94	t06B	MX	Clinical
MM-104	t13	MX	Clinical
MM-107	t13	MX	Clinical
MM-102	t13	MX	Clinical
MM-34	t14	MX	Clinical
MM-01	t04A	AR	Environmental
MM-03	t03	AR	Environmental
MM-14	t01	AR	Clinical
MM-77	t01	AR	Clinical
MM-18	t02	AR	Clinical
MM-95	t02	AR	Clinical
MM-26	t02	AR	Clinical
MM-05	t03	AR	Clinical
MM-06	t03	AR	Clinical
MM-17	t03	AR	Clinical
MM-86	t03	AR	Clinical
MM-84	t03	AR	Clinical
MM-21	t03	AR	Clinical
MM-23	t03	AR	Clinical
MM-12	t04A	AR	Clinical
MM-15	t04A	AR	Clinical
MM-20	t04A	AR	Clinical
MM-97	t04A	AR	Clinical
MM-78	t04A	AR	Clinical
MM-79	t04A	AR	Clinical
MM-24	t04A	AR	Clinical
MM-25	t04A	AR	Clinical
MM-22	t04A	AR	Clinical
MM-81	t04A	AR	Clinical
MM-19	t04A	AR	Clinical
MM-96	t14	AR	Clinical
MM-75	t25 *	AR	Clinical
MM-13	t26 *	AR	Clinical
MM-16	t26 *	AR	Clinical
MM-55	t03	FR	Clinical
MM-51	t04A	FR	Clinical
MM-52	t04A	FR	Clinical
MM-56	t04A	FR	Clinical
MM-61	t04A	PE	Clinical
MM-62	t01	PE	Clinical
MM-63	t01	PE	Clinical

* indicates new CSP types; MX: Mexico; AR: Argentina; FR: France; PE: Peru.

**Table 2:** Reference sequences corresponding to the *benA*.

Access Number	Species GenBank
MG991419.1	*A. fumigatus*
MG991417.1	*A. fumigatus*
KT253231.1	*A. lentulus*
KX903289.1	*A. lentulus*
MH614456.1	*A. niger*
MH614419.1	*A. neoniger*
MH277121.1	*A. luchuensis*
MH614561.1	*A. luchuensis*
MH204832.1	*A. niger*
KC433673.1	*A. acidus*
MF150885.1	*A. acidus*
KJ469441.1	*A. piperis*
KC796359.1	*A. piperis*
MH494188.1	*A. niger*
KR064615.1	*A. welwitschiae*
MH208740.1	*A. welwitschiae*
MH614546.1	*A. costaricaensis*
MK119733.1	*A. flavus*
MK119732.1	*A. flavus*
MH279881.1	*A. oryzae*
MH279871.1	*A. oryzae*
KP329874.1	*A. westerdijkiae*
KP329872.1	*A. westerdijkiae*
DQ768444.1	*A. foetidus*
FJ828925.1	*A. foetidus*

**Table 3:** Summary of repeat type sequences identified on the cell surface protein (CSP).

r01	ACT TCT GTC CCG
r02	ACT TCT GTC CCA
r03	ACT CAA AAC GCG
r04	ACT TCA ATC CCG
r05	ACT TTT GTC CCG
r06	ACT TCA GTC CCG
r07	ACT ACT ATT GTG
r08	ACT TTT CTC CCG
r09	ACT TCT GTT CCG
r10	ACT TCA ATC CCA

**Table 4:** CSP types identified to date among *A. fumigatus* isolates.

CSP	Codon	Tandem Repeat Succession	Codon
Type	−15 −14 −1		+1 +2 +3
t01	GTG GTC CCG	01-01-01-01-05-03-01-06-03-07	CCA CCT
t02	GTG GTC CCG	01-01-02-03-04-05-03-01-06-03-07	CCA CCT
t03	GTG GTC CCG	01-02-03-04-06-03-07	CCA CCT
t04A	GTG GTC CCG	01-02-03-04-05-03-01-06-03-07	CCA CCT
t04B **	GTG GTC CCA	01-02-03-04-05-03-01-06-03-07	CCA CCT
t05 **	GTG GTC CCG	01-01-01-03-01-06-03-07	CCA CCT
t06A **	GTG GTC CCG	01-01-01-02-03-04-05-03-01-06-03-07	CCA CCT
t06B	GTG CTC CCG	01-01-01-02-03-04-05-03-01-06-03-07	CCG CCT CCT
t07 **	GTG CTC CCG	01-02-03-04-05-03-04-05-03-01-06-03-07	CCG CCT CCT
t08 **	GTG CTC CCG	01-01-01-02-03-04-05-03-04-05-03-01-06-03-07	CCG CCT CCT
t09 **	GTG GTC CCG	01-01-01-01-01-05-03-01-06-03-07	CCA CCT
t10	GTG GTC CCG	01-01-01-05-03-01-06-03-07	CCA CCT
t11 **	GCG CTC CCG	01-01-08-03-01-06-03-07	CCA CCT
t12 **	GTG GTC CCG	01-01-01-01-01-02-03-04-05-03-01-06-03-07	CCA CCT
t13	GTG CTC CCG	01-01-02-03-04-05-03-04-05-03-01-06-03-07	CCG CCT CCT
t14	GTG GTC CCG	01-01-01-01-02-03-04-05-03-01-06-03-07	CCA CCT
t15 **	GTG CTC CCG	01-01-01-01-02-03-04-05-03-04-05-03-01-06-03-07	CCG CCT CCT
t16 **	GTG GTC CCG	01-05-03-01-06-03-07	CCA CCT
t17 **	GTG GTC CCG	01-01-02-03-04-05-03-09-06-03-07	CCA CCT
t18A **	GTG GTC CCG	01-01-05-03-01-06-03-07	CCA CCT
t18B **	GCG CTC CCG	01-01-05-03-01-06-03-07	CCA CCT CCA
t19 **	GTG CTC CCG	01-01-02-03-10-03-04-05-03-04-05-03-01-06-03-07	CCG CCT CCT
t20 **	GTG GTC CCG	01-02-03-07	CCA CCT
t21 **	GTG GTC CCG	01-02-03-04-04-03-07	CCA CCT CCA
t22 **	GTG GTC CCG	01-01-02-03-01-06-03-07	CCA CCT CCA
t23 **	GTG GTC CCG	01-01-02-03-04-05-03-07	CCA CCT CCA
t24 **	GTG GTC CCG	01-01-01-01-03-01-06-03-07	CCA CCT CCA
t25 *	GTG GTC CCG	01-01-02-03-04-05-03-01-01-03-07	CCA CCT
t26 *	GTG GTC CCG	01-06-03-07	CCA CCT

The table shows all the CSP types reported so far by other authors [19,20,21,22]. * CSP types not reported among isolates in previous studies. ** CSP types not observed among the México, Argentina, France, and Peru isolates in this study.

**Table 5:** Prevalence of CSP types in *A. fumigatus* isolates from Mexico, Argentina, France, and Peru.

	Mexico			Argentina			France	Peru
CSP Type	Clin (*n* = 28)	Env (*n* = 26)	Overall (54)	Clin (*n* = 27)	Env (*n* = 2)	Overall (*n* = 29)	Clin (*n* = 4)	Clin (*n* = 3)
t01	14.2%	11.5%	12.9%	7.4%		6.8%		66.6%
t02	14.2%	11.5%	12.9%	11.1%		10.3%		
t03	3.5%	11.5%	7.4%	25.9%	50%	27.5%	25%	
t04A	25%	53.8%	38.8%	40.7%	50%	41.3%	75%	33.3%
t06B	28.5%		14.8%					
tT10		11.5%	5.5%					
t13	10.7%		5.5%					
t14	3.5%		1.8%	3.7%		3.4%		
t25 *				3.7%		3.4%		
t26 *				7.4%		6.8%		

Clin, clinical isolate; Env, environmental isolate; * CSP type t25 and t26 are new types.

**Table 6:** Parameters of genetic diversity in populations of *A. fumigatus.*

Population	*n*	S	Hd	Pi	K	Tajima’s D
MX	54	168	0.788 ± 0.029	0.02235 ± 0.00925	9.029	−2.8372 (*p* < 0.001)
AR	28	18	0.823 ± 0.052	0.00524 ± 0.00148	2.312	−2.02849 (*p* < 0.05)
FR	4	2	0.500 ± 0.265	0.00190 ± 0.00101	1.000	−0.70999 (*p* > 0.10)

*n*, number of sequences; S, variable sites; Hd, haplotype diversity; Pi, average number of nucleotide differences per site between two sequences chosen at random; K, average number of nucleotide differences between pairs of sequences.

## Supplementary Materials

The following are available online at https://www.mdpi.com/2076-2607/8/5/688/s1, Figure S1: Molecular phylogenetic analysis by Maximum Likelihood method.
