# Supplementary material for: Identification of CSP Types and Genotypic Variability of Clinical and Environmental Isolates of Aspergillus fumigatus from Different Geographic Origins
Source: Microorganisms. 2020 May 8;8(5):688. doi: 10.3390/microorganisms8050688 (PMC7284390; doi:10.3390/microorganisms8050688)
Supplement: Supplementary file 1 [file microorganisms-08-00688-s001.pdf]

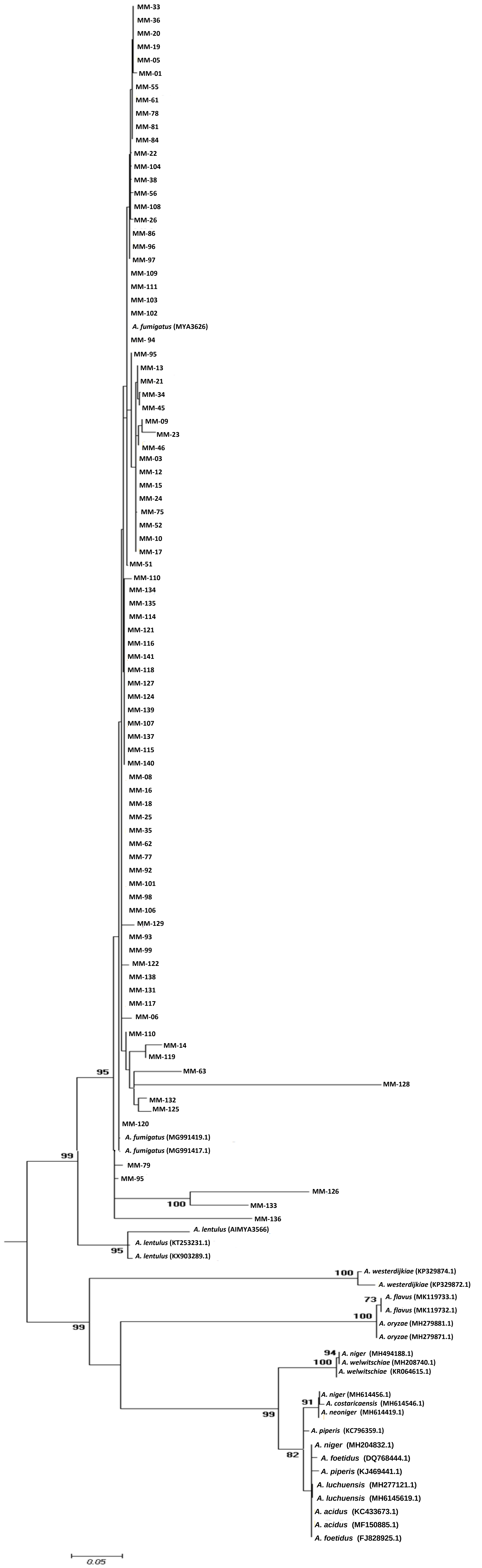

Figure S1. Molecular phylogenetic analysis by Maximum Likelihood method. The evolutionary history was inferred by using the Maximum Likelihood method based on the General Time Reversible model. The bootstrap consensus tree inferred from 1000 replicates is taken to represent the evolutionary history of the taxa analyzed.
